# Association between Post-Transplant Vitamin D, Metabolic Syndrome, and Post-Transplant Diabetes Mellitus

**DOI:** 10.34067/KID.0000000763

**Published:** 2025-03-07

**Authors:** Rohit Malyala, Karan Vansjalia, Michelle Nash, Niki Dacouris, Lindita Rapi, G.V. Ramesh Prasad

**Affiliations:** 1 Kidney Transplant Program, St. Michael's Hospital, Toronto, Ontario, Canada; 2 Division of Nephrology, Department of Medicine, University of Toronto, Toronto, Ontario, Canada

**Keywords:** diabetes, kidney transplantation, vitamin D

## Abstract

**Key Points:**

- Metabolic syndrome components after kidney transplant are associated with a higher likelihood of post-transplant diabetes.
- This predictive association persists independently of other prediabetes markers, including fasting sugar and hemoglobin A1c.
- Higher vitamin D-25 levels blunt this association; in patients with high metabolic syndrome burden, vitamin D supplementation may be warranted.

**Background:**

Associations between 25-hydroxyvitamin D (25(OH)D) deficiency and diabetes have been observed in the general population, but are less delineated in kidney transplant recipients (KTRs), especially in the context of highly prevalent metabolic syndrome (MetS) features in KTRs. We hypothesized that vitamin D deficiency may present greater risk in KTRs with greater burden of MetS features.

**Methods:**

We retrospectively evaluated 1792 KTRs with no treated diabetes at transplant between 1998 and 2018. Vitamin D was measured at ≥3 months post-transplant. MetS features were defined by the National Cholesterol Education Program, Adult Treatment Panel III (NCEP-ATP-III) criteria. The primary outcome was treated post-transplant diabetes mellitus (PTDM) incidence.

**Results:**

In 1792 nondiabetic KTRs followed for 10,956 patient-years, 237 patients developed PTDM. For KTRs meeting NCEP-ATP-III criteria with fourth-quartile 25(OH)D, there were 1.5 new diagnoses per 100 patient-years versus 4.2 events per 100 patient-years in KTRs with first-quartile 25(OH)D (*P* < 0.001). In multivariate survival regression, vitamin D was, accounting for individual NCEP-ATP-III criteria, associated with PTDM (hazard ratio, 0.93 per 10 nmol/ml 25(OH)D, *P* = 0.007) independently of fasting blood sugar and hemoglobin A1c. In marginal effects analysis, MetS effect on PTDM increased as serum 25(OH)D levels decreased.

**Conclusions:**

Our study suggests that decreased 25(OH)D is associated with increased PTDM, and this marginal effect worsens as KTRs have an increased burden of MetS.

## Visual Abstract

**Figure:**
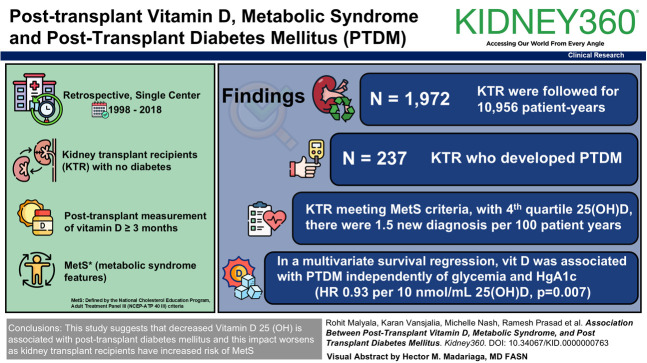

## Introduction

Kidney transplantation is the gold standard treatment for ESKD, offering improved quality of life and increased survival rates for patients.^1,2^ However, despite advancements in transplantation techniques and immunosuppressive therapies, post-transplant diabetes mellitus (PTDM) remains a significant cause of morbidity and mortality among kidney transplant recipients (KTRs).^3^

Among the potential contributors to adverse outcomes in KTRs, 25-hydroxyvitamin D (25(OH)D) deficiency has emerged as an interesting, potentially modifiable candidate. 25(OH)D plays a pivotal role in various physiologic processes beyond its classical role in calcium homeostasis, including immune modulation, endothelial function, and regulation of the renin-angiotensin-aldosterone system.^4^ 25(OH)D deficiency is highly prevalent among KTRs, likely because of impaired 25(OH)D synthesis, increased catabolism, and limited exposure to sunlight.^5^

The association between 25(OH)D deficiency, metabolic syndrome (MetS), and diabetes incidence has been extensively studied in the general population, highlighting its potential role in the development and progression of MetS and subsequent PTDM.^6,7^ Data also exist on 25(OH)D deficiency and MetS components on PTDM and graft survival individually of each other.^8,9^ However, limited data exist specifically addressing the effect of post-transplant vitamin D status and the co-occurrence of MetS features and their influence on the incidence of PTDM in KTRs.

The primary objective of this retrospective cohort study was hence to examine the associations between post-transplant 25(OH)D and co-occurrence of features of MetS, and marginal effects on a primary outcome of PTDM incidence. This study was performed in a KTR population that was free of treated diabetes at the time of transplant and at the time of 25(OH)D measurement post-transplant and with the prevalence of MetS features being determined at the time of 25(OH)D measurement.

## Methods

### Study Sample and Follow-Up

The study was conducted at St. Michael's Hospital (SMH), which is an urban university-affiliated medical-surgical center specializing in kidney transplantation. The hospital actively monitors a cohort of over 1700 KTRs and performs approximately 130 single-organ kidney transplants on adult patients each year. Follow-up visits are scheduled at regular intervals post-transplant, ranging from weekly to annually, during which trained staff record anthropometric measurements and resting BP. Patients self-report their sex and ethnicity, and laboratory tests were conducted close to each clinic visit, with additional testing performed on a separate, more frequent schedule. The eGFR is calculated using the Modification of Diet in Renal Disease-7 equation.^10^

In this study, we identified a cohort not treated for diabetes who were transplanted and followed at SMH between September 1, 1998, and December 31, 2018, with a serum 25(OH)D level drawn at or beyond 3 months of transplant, extracting records from the SMH clinical patient data management system. No specific or centralized laboratory was used for 25(OH)D blood tests. No other exclusion criteria were applied. Clinical data relevant to the diagnosis of MetS were cross-sectionally obtained from time points closest to vitamin D-25 measurement. Follow-up for PTDM incidence and PTDM-free survival began at the time of vitamin D-25 measurement. Patients with formally diagnosed diabetes (diabetic nephropathy) or receiving treatment with oral hypoglycemics or insulin, at the time of transplant or at the time of vitamin D-25 measurement, were excluded from the study.

The study obtained approval from the Institutional Research Ethics Board (REB-10-204). As this study involved a retrospective review of clinic data pertaining to a prevalent KTR population, individual informed consent was not obtained. The study was conducted in accordance with the ethical principles outlined in the 2000 Declaration of Helsinki and the 2008 Declaration of Istanbul.

### Patient Evaluation and Data Collection

PTDM diagnoses were regularly recorded through transcriptions from local and citywide hospital databases as part of routine patient care. In cases where additional information was needed, interviews with patients and their family physicians were conducted to supplement the data. The initiation or use of medications, including oral hypoglycemics and insulin, was longitudinally referenced through chart review and/or patient data management systems at SMH for determining PTDM-free survival.

In this study, patients were classified as having MetS on the basis of the National Cholesterol Education Program, Adult Treatment Panel III (NCEP-ATP-III) definition.^11^ According to the NCEP-ATP-III, MetS is considered present when three or more of the following five criteria are met:

1. Waist circumference exceeding 40 inches (102 cm) for men or 35 inches (88 cm) for women.
2. BP higher than 130/85 mm Hg.
3. Fasting triglyceride (TG) level exceeding 150 mg/dl (1.69 mmol/L) or TG-lowering medication use.
4. Fasting HDL cholesterol level below 40 mg/dl for men or 50 mg/dl for women (1.03 and 1.29 mmol/L, respectively) or lipid-lowering medication use.
5. Fasting blood glucose level over 100 mg/dl (5.6 mmol/L) or hypoglycemic medication use.

Fulfillment of these criteria was considered on the basis of patient status at the relevant blood draws and encounters nearest to the 25(OH)D bloodwork date. 25(OH)D in this study is analyzed as a continuous and categorized variable. When categorized, cutoffs for vitamin D subgroups were based on quartiles or median.

### Statistical Analyses

The statistical analyses were conducted using R, version 4.2.0. Reported data are presented as mean and SD for numeric variables or mean and percentage for categorical variables, unless otherwise specified. Statistical significance was determined using two-tailed hypothesis tests.

Missing values in the dataset were handled by exclusion from the relevant analyses without imputation. The appropriate statistical tests were used for comparisons, including *t* test, ANOVA, chi-squared test, or Cox proportional hazards analysis, depending on the nature of the data, including tests for trends in proportions for data with ordinal independent variables. Survival curves are constructed based on Kaplan-Meier estimates with corresponding *P* values based on the log-rank test. To assess the normality and skewness of subgroups for relevant analyses, visual inspection with skewness testing was used (not shown). In analyses depending on categorization of 25(OH)D levels, quartiles or medians have been used as we have found poor agreement in the literature on definitive levels of normal/deficient vitamin D levels.

In marginal effects analysis using Cox regression models, selected covariates were chosen based on biologic and clinical relevance, including sex, age, fasting blood sugar, and hemoglobin A1c (HbA1c) at the time of 25(OH)D measurement, in addition to 25(OH)D and MetS burden, as measured by the number of NCEP-ATP-III criteria. Schoenfeld residuals plots and *P* values were inspected to ensure the proportional hazards assumption in univariate or multivariate Cox models. Variable measurements that were derived from recordings nearest to the time of vitamin D-25 bloodwork were used exclusively in all analyses, as event incidence and risk were measured from the time of vitamin D bloodwork post-transplant and not from the time of transplant itself.

## Results

### Pretransplant and Post-Transplant Characteristics across MetS and Vitamin D Status

Data were available on 2136 KTRs transplanted at SMH between 1998 and 2018, with 344 KTRs excluded from analysis on the basis of formally diagnosed or treated diabetes before or at the time of 25(OH)D measurement. A total of 1792 eligible KTRs were hence enrolled in the study, providing a total of 10,956 years of patient follow-up. Table 1 presents baseline patient characteristics at 3 months post-transplant. Patients are grouped into four categories for comparisons: with and without MetS and above or below median levels of serum vitamin D-25. In total, 191 cases of new post-bloodwork PTDM are recorded over the follow-up interval for an incident rate of 10.66% (191/1792) or 1.743 events per 100 patient-years. The vitamin D-25 median in all comers was 48.0 (interquartile range, 38.0–68.0; mean [SD], 54.3 [27.4]). No statistically significant difference was found between groups in ANOVA with age at vitamin D bloodwork (*P* = 0.125). Average time to bloodwork from transplant was 2.81 years. At the time of vitamin D measurement, parathyroid hormone (PTH) levels were notably higher in patients with below median vitamin D (above median 25(OH)D PTH: 11.4±11.5 versus below median 25(OH)D PTH: 17.9±20.5, *P* < 0.001). Random blood glucose levels were also higher in KTRs with below median 25(OH)D regardless of MetS status (above median 25(OH)D: 6.63±3.7 mmol/L versus below median 25(OH)D: 7.19±3.6, *P* < 0.001).

**Table 1:** Baseline recipient characteristics and summary statistics

	No MetS		MetS		*P* Value	Missing
Vitamin D Status	Below Median	Above Median	Below Median	Above Median		
*n*	286	346	594	566		
**Patient demographics**						
Age at transplant (yr)	50.12 (14.22)	47.89 (13.22)	50.54 (13.71)	51.03 (13.73)	0.007^a^	0
Age at bloodwork (yr)	52.98 (13.78)	52.71 (12.97)	52.50 (13.32)	54.25 (13.17)	0.125	0
Mean years follow-up	5.69 (4.38)	8.58 (5.14)	4.58 (3.81)	6.43 (4.72)	<0.001^a^	0
Ethnicity					0.001^a^	0
*Black/Afro-Caribbean*	17 (5.9)	12 (3.5)	43 (7.2)	26 (4.6)		
*East Asian*	36 (12.6)	29 (8.4)	72 (12.1)	49 (8.7)		
*Other/unknown*	157 (54.9)	205 (59.2)	329 (55.4)	349 (61.7)		
*South Asian*	23 (8.0)	17 (4.9)	42 (7.1)	16 (2.8)		
*White*	53 (18.5)	83 (24.0)	108 (18.2)	126 (22.3)		
**Patient characteristics**						
Systolic BP (mm Hg)	128.58 (15.09)	127.65 (15.12)	128.25 (15.98)	128.62 (15.48)	0.848	11.5
Diastolic BP (mm Hg)	80.35 (10.13)	81.19 (9.61)	80.83 (10.44)	80.33 (9.74)	0.625	11.5
BMI (kg/m^2^)	27.46 (12.87)	27.12 (12.05)	26.44 (10.50)	27.62 (22.21)	0.664	14.0
Waist-to-hip ratio	0.96 (0.07)	0.99 (0.75)	0.99 (0.57)	0.95 (0.08)	0.396	19.0
Smoking status (%)	87 (34.5)	127 (41.8)	205 (37.5)	203 (40.5)	0.255	10.5
Male sex (%)	167 (58.4)	203 (58.8)	365 (61.6)	344 (60.8)	0.757	0.1
Live donor (%)	176 (61.5)	151 (43.6)	363 (61.1)	278 (49.1)	<0.001^a^	0.0
Pretransplant MACE (%)	47 (16.4)	47 (13.6)	87 (14.6)	96 (17.0)	0.491	0.0
Acute rejection	17 (5.9)	9 (2.6)	45 (7.6)	27 (4.8)	0.010^a^	0
Delayed graft function	11 (3.8)	9 (2.6)	26 (6.1)	18 (3.2)	0.030^a^	0
**3-mo post-transplant bloodwork**						0
HDL (mmol/L)	1.31 (0.48)	1.33 (0.44)	1.33 (0.46)	1.42 (1.03)	0.087	
LDL (mmol/L)	2.64 (1.10)	3.12 (9.75)	2.51 (0.93)	2.67 (1.01)	0.297	8.4
Non-HDL cholesterol (mmol/L)	3.51 (1.44)	3.37 (1.05)	3.44 (2.01)	3.45 (1.47)	0.764	14.3
TGs (mmol/L)	1.95 (1.38)	1.83 (1.02)	1.89 (1.04)	1.80 (1.07)	0.288	8.4
24-h urine protein	0.86 (2.26)	0.50 (0.85)	0.82 (1.47)	0.60 (1.17)	0.046	5.8
Fasting blood gluc. (mmol/L)	6.20 (2.25)	5.80 (1.79)	6.38 (4.92)	6.15 (5.17)	0.255	56.8
Random blood gluc. (mmol/L)	6.63 (4.09)	6.01 (2.03)	6.57 (2.67)	6.30 (3.91)	0.039^a^	0.9
Apolipoprotein A1	1.49 (0.32)	1.50 (0.27)	1.50 (0.32)	1.55 (0.28)	0.050^a^	0.4
Apolipoprotein B	0.83 (0.24)	0.84 (0.23)	0.84 (0.25)	0.83 (0.23)	0.946	22.9
Apo-B/Apo-A1 ratio	0.58 (0.20)	0.58 (0.19)	0.58 (0.24)	0.55 (0.18)	0.166	22.5
Serum creatinine (umol/L)	133.79 (92.79)	128.14 (74.87)	130.57 (64.94)	129.98 (69.35)	0.810	23.2
GFR (MDRD-7)	60.78 (77.30)	53.89 (18.04)	54.54 (21.28)	67.18 (261.81)	0.457	0
C-reactive protein (mg/L)	8.64 (25.19)	6.49 (14.80)	6.70 (20.18)	6.49 (15.57)	0.401	0
Albumin-to-creatinine ratio (mg/mmol)	16.69 (59.11)	9.82 (32.30)	10.41 (26.96)	9.37 (23.55)	0.023^a^	0.4
Uric acid (mmol/L)	392.55 (115.41)	393.11 (106.43)	388.95 (111.74)	388.99 (100.50)	0.929	2.5
PTH (ng/ml)	19.09 (22.14)	11.23 (11.97)	17.85 (20.25)	11.84 (12.00)	<0.001^a^	14.2
Vitamin D-25 (nmol/ml)	32.26 (10.01)	76.73 (23.06)	31.67 (10.05)	72.91 (20.96)	<0.001^a^	0.3
**Prescriptions at 3 mo**						0
Tacrolimus (versus cyclosporine)	168 (75.0)	196 (75.7)	345 (81.8)	332 (81.0)	0.079	
Statin (%)	137 (52.9)	147 (50.2)	253 (46.5)	276 (55.5)	0.030^a^	11.1
ACE-inhibitor (%)	49 (18.9)	72 (24.6)	62 (11.4)	100 (20.1)	<0.001^a^	11.1
ARB (%)	49 (18.9)	71 (24.2)	92 (16.9)	97 (19.5)	0.087	11.1
Oral hypoglycemic (%)	38 (14.7)	29 (9.9)	63 (11.6)	56 (11.3)	0.356	11.1
Prednisone (%)	197 (87.9)	220 (84.9)	376 (89.1)	366 (89.3)	0.332	26.6

Criteria for metabolic syndrome are based on National Cholesterol Education Program, Adult Treatment Panel III criteria. “Above median” vitamin D-25 level is >48 nmol/ml. Mean and (SD) are provided for continuous variables. Binary variables are indicated as count and (percentage). ACE, angiotensin-converting enzyme; ARB, angiotensin receptor blocker; BMI, body mass index; MACE, major adverse cardiac events; MetS, metabolic syndrome; MDRD-7, modification of diet in renal disease-7 (a study name); PTH, parathyroid hormone; TG, triglyceride.

a *P* < 0.05.

### MetS Status and Vitamin D Status and PTDM Incidence

In KTRs not meeting NCEP-ATP-III criteria for MetS, fourth-quartile 25(OH)D (high vitamin D) patients had 1.8 new diagnoses of PTDM per 100 patient-years (years post-bloodwork free of event), whereas first-quartile 25(OH)D patients had 3.4 events per 100 patient-years (*P* = 0.011, all *P* values reporting chi-squared test for trend in proportions). In KTRs with MetS, for patients with fourth-quartile 25(OH)D, there were 1.5 new diagnoses per 100 patient-years of follow-up versus 4.2 new diagnoses per 100 patient-years in KTRs with first-quartile 25(OH)D (*P* < 0.001).

**Figure 1:**
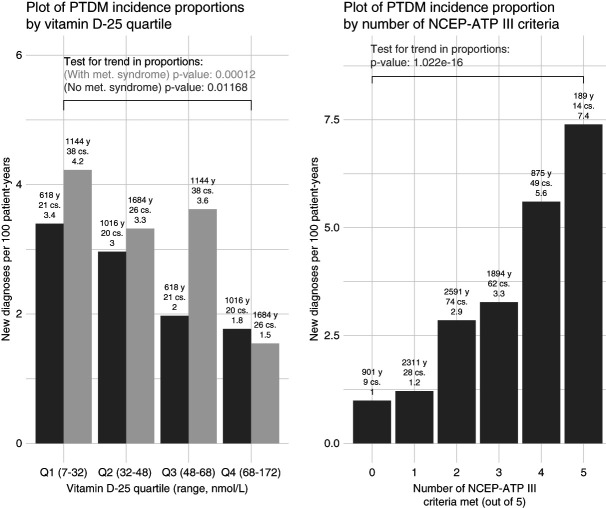
**PTDM incidence proportions across quartiles of vitamin D and number of criteria for MetS met.** Criteria for MetS are based on NCEP-ATP-III criteria. Hypothesis testing conducted through the chi-squared test for trends in incidence proportions. Years of follow-up, case counts, and event counts per 100 patient-years are included in annotations above individual bars. MetS, metabolic syndrome; NCEP-ATP-III, National Cholesterol Education Program, Adult Treatment Panel III; PTDM, post-transplant diabetes mellitus.

Similarly, PTDM incidence rose with increased MetS burden, categorized by the number of features of MetS carried by each KTR 3 months post-transplant. KTRs with zero NCEP-ATP-III criteria had 1.00 new diagnoses per 100 patient-years, whereas KTRs with all five criteria had 7.4 new diagnoses per 100 patient-years (*P* < 0.001; Figure 1).

### Features of MetS in Association with PTDM Incidence

**Figure 2:**
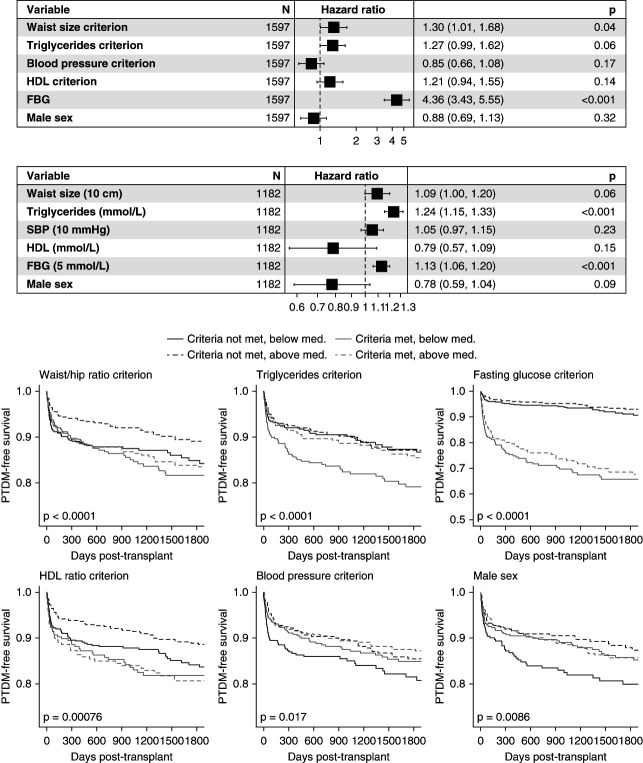
**Features of the NCEP-ATP-III definition of MetS and association in multivariate logistic regression with PTDM survival.** Forest plots demonstrating hazard ratios of various components of the NCEP-ATP-III definition of MetS in multivariable regression. Samples where all columns of data (MetS criteria) were not fully accounted for were excluded from the analysis. The upper forest plot uses binary variables based on the NCEP-ATP-III criteria (*e.g*., BP criterion is met, 0=no, 1=yes). The lower forest plot uses continuous versions of the relevant variables from the definition. Below, Kaplan-Meier survival curves demonstrate univariate impacts of each of the criteria on PTDM-free survival. Solid line=below median 25(OH)D, dashed line=above median 25(OH)D; black line=does not meet MetS criteria, gray line=does meet MetS criteria. Note the *y* axis range of the KM plot for fasting glucose is wider than other plots, and spans from 0.5 to 1.0. 25(OH)D, 25-hydroxyvitamin D. FBG, Fasting blood glucose; KM, Kaplan-Meier; SBP, systolic blood pressure.

In Figure 2, the five criteria of the NCEP-ATP-III definition of MetS, as well as a sixth modifying criterion of sex, were included in multiple Cox regression, as standalone criteria (top forest plot) and as continuous variables (bottom forest plot). Expressed as criteria, only waist-to-hip ratio (hazard ratio [HR], 1.30; *P* = 0.04) and fasting blood glucose (HR, 4.36 per each mmol/L; *P* < 0.001) were statistically significantly associated with PTDM-free survival. Expressed as continuous criteria, serum TGs were also statistically significant (HR, 1.25 per mmol/L; *P* < 0.001). All criteria were also expressed as univariate Kaplan-Meier survival plots, with stratification by above/below median 25(OH)D status. In univariate plots, criteria using the waist-to-hip ratio, TGs, fasting glucose, and HDL were all statistically significant at *P* < 0.05. 25(OH)D notably had variable effects on the hazard associated with meeting different MetS criteria in univariate Kaplan-Meier plots. The predictive effect of each criterion was often differentially more marked in either the above median group (*e.g*., HDL: low 25(OH)D in high HDL versus low HDL *P* = 0.75, versus high 25(OH)D in high HDL versus low HDL *P* = 0.004) or in the below median group (*e.g*., TGs: low 25(OH)D in high TG versus low TG *P* = 0.003, versus high 25(OH)D in high HDL versus low HDL *P* = 0.035; *P* values derived from pairwise tests from Kaplan-Meier curves).

### Kaplan-Meier and Multiple Cox Regression for PTDM-Free Survival

**Figure 3:**
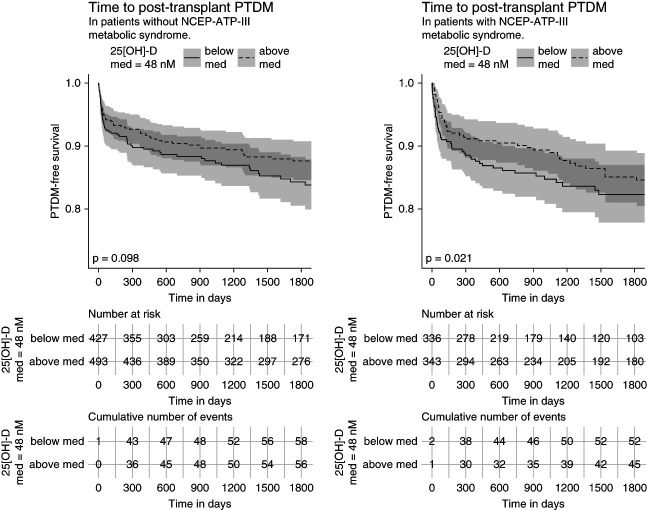
**Kaplan-Meier survival curves for PTDM incidence across vitamin D median levels.** Criteria for MetS are based on NCEP-ATP-III criteria. Vitamin D-25 median in this cohort is 48 nmol/ml. Risk tables and cumulative events tables are included below for each KM plot, respectively.

Kaplan-Meier curves showing PTDM-free survival are shown for patients with above median and below median values of 25(OH)D with and without MetS (Figure 3). In KTRs with MetS, there is a statistically significant difference in 5-year survival between 25(OH)D strata, with increased 25(OH)D being protective (*P* = 0.021). In KTRs without MetS, the same trend holds, but falls below threshold for statistical significance (*P* = 0.098).

### Marginal Effects of MetS Criteria on Association of Vitamin D and PTDM

**Figure 4:**
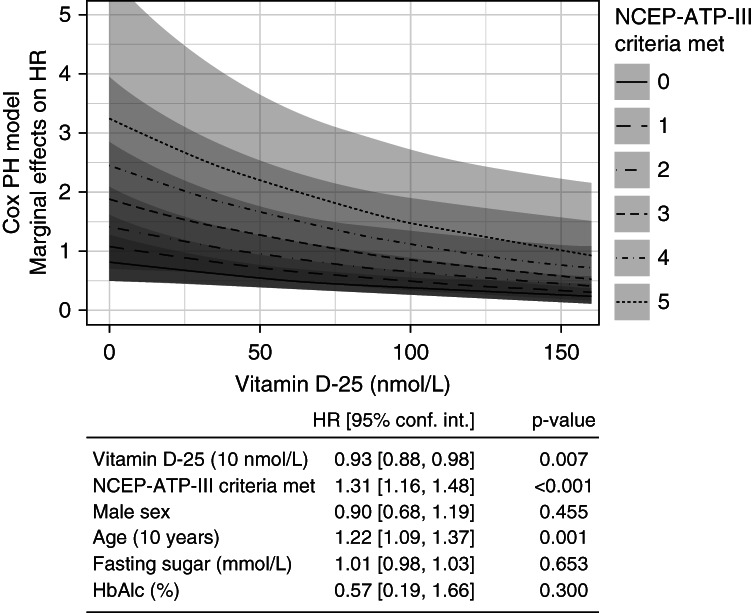
**Multiple Cox regression and marginal effects plot for PTDM incidence across vitamin D level and number of MetS criteria met.** Plot demonstrating the marginal effects on estimated hazard ratios from a Cox survival regression of the effects of vitamin D-25 on downstream PTDM survival at different levels of the MetS, based on criteria of the MetS met at the time of bloodwork. No interaction terms are used in this regression model. Table containing HR estimates with confidence intervals and *P* values is included for the depicted model. HbA1c, hemoglobin A1c; HR, hazard ratio; PH, proportional hazards.

In Figure 4, a marginal effects plot is constructed to show the effects of increased MetS burden, represented by the number of NCEP-ATP-III criteria, on the HRs of low 25(OH)D for PTDM incidence. The multivariate Cox model represented also contains four other covariates selected on the basis of clinical relevance, including patient sex, age, fasting glucose, and HbA1c at the time of 25(OH)D bloodwork. Both 25(OH)D and MetS burden were statistically significantly associated with PTDM survival in the model (25(OH)D per 10 mmol/L HR: 0.93, *P* = 0.007, NCEP-ATP-III criteria count HR: 1.31, *P* < 0.001). In the plot, the effect of MetS burden seemed to be magnified at lower serum 25(OH)D levels.

Fasting glucose and HbA1c trended toward, but did not achieve, statistical significance for prognosticating PTDM-free survival, when included in this Cox model with 25(OH)D and MetS burden (*P* = 0.653 and 0.300, respectively). In a separately conducted interaction terms analysis, the formal interaction term between 25(OH)D and MetS burden was statistically significant (HR, 1.08 [1.03–1.13], *P* = 0.002, figure not shown).

## Discussion

We had hypothesized that lower cross-sectional serum 25(OH)D measurements post-transplant would be associated with subsequent PTDM incidence, especially as the burden of MetS increased in individual KTRs. We found that low 25(OH)D levels seemed to indeed be associated with an increased relative hazard of MetS burden on PTDM survival or *vice versa*.

Clinically, there is high prevalence of MetS, the components thereof in pretransplant and post-transplant KTRs.^12^ The previous literature has linked MetS to PTDM and PTDM and glucose intolerance to long-term adverse outcomes in KTRs.^13–15^ The effects of MetS on renal injury are likely multifactorial, including insulin resistance and oxidative stress, heightened production of proinflammatory cytokines, augmented microvascular damage and subsequent renal ischemia, and more.^16^ The interplay between MetS and CKD is complex and likely bidirectional.^16^ The post-transplant milieu, especially with respect to the heavy immunosuppression and other medication burden, exposes patients to additional pathophysiology, resulting in further graft injury. For example, corticosteroids increase resistance of the liver, muscles, and peripheral tissues to insulin and decreases glucose uptake; calcineurin inhibition decreases intracellular nuclear transcription factors that promote insulin gene transcription.^15^

However, before the International Consensus Guidelines in 2003,^12^ there was no consistent definition for PTDM, and there has been little consistency between centers for diabetes screening pretransplant as well. Numerous early studies on PTDM likely included individuals broadly without accounting for undetected or untreated diabetes and MetS before transplantation.^12,14^ Since the International Consensus Guidelines in 2003, significant advances in diabetes mellitus (DM) management in the general population have prompted the third international PTDM Consensus Meeting in 2024.^17^ However KTR-specific evidence was recognized to be sparse, and as such, the 2024 update includes opinion statements exclusively concerning optimal diagnostic methods and timing of assessments and means of PTDM prevention. The 2003 statement and 2024 guidelines alike indicate that patients should be screened pretransplant for MetS features as a known determinant of PTDM. The consideration of comorbid factors is an important step in the clinical assessment of patients pretransplant and is recommended to be used to individualize therapy and pre/post-transplant care, including potential vitamin D-25 supplementation.

The potential effects of vitamin D on insulin secretion and insulin resistance are pleiotropic and supported broadly in the laboratory and clinical literature.^18^ Vitamin D receptors are expressed in pancreatic *β*-cells,^19^ and vitamin D responsive elements have been identified in human gene promoters encoding for insulin.^20^ Clinically, in nontransplant populations, 25(OH)D levels are inversely correlated with DM prevalence,^21^ with 25(OH)D insufficiency being associated with insulin resistance and increased HbA1c.^21,22^

In KTRs specifically, the effect of MetS on PTDM has previously been investigated in smaller prospective and retrospective studies.^8^ Separately, 25(OH)D deficiency has been identified as an independent risk factor of PTDM in kidney transplant.^9^ The recent Vitamin D Supplementation in Renal Transplant Recipients Randomized Controlled Trial^23^ did not demonstrate improvement in nonskeletal complications of kidney transplant with vitamin D supplementation, including PTDM, nor improvement in HbA1c levels. However, MetS is a cluster of several modifiable factors outside of HbA1c; vitamin D deficiency may have marginally different effects when comorbid with the syndrome, especially in diverse multiethnic cohorts.^24,25^ Furthermore, the Vitamin D Supplementation in Renal Transplant Recipients study considered PTDM incidence as part of a composite outcome and was underpowered for assessment of individual components of the composite.

The clinical relevance of our findings is two-fold. First, our results highlight the importance of assessing and addressing vitamin D status in KTRs, particularly those with MetS. Vitamin D deficiency is prevalent in this population, and our findings suggest that correcting this deficiency may be particularly crucial, especially in individuals with MetS, to mitigate the risk of new-onset diabetes. Routine monitoring of serum 25(OH)D levels and targeted interventions, such as supplementation, may be warranted in these patients.

Second, our findings underscore the potential role of 25(OH)D as a potential modulating factor in the relationship between other MetS features and diabetes after kidney transplantation. Supporting this hypothesis, multivariate analysis with an interaction term for 25(OH)D by MetS criteria indicated positive interaction with a multiplicative effect of these variables (HR, 1.08 [1.03–1.13]; *P* = 0.002). However, although the interaction term supports the hypothesis, the follow-up time was not sufficient for more dedicated interactions testing especially at higher MetS burdens, and the analysis focuses on impacts for marginal effects. Furthermore, not all MetS features seem to represent the same risk for developing PTDM. The previous study in post-transplant major adverse cardiac events has demonstrated that different components of MetS have differential impacts post-transplant major adverse cardiac events.^24^

This cohort study is limited by its retrospective nature and should be considered hypothesis generating rather than capable of establishing causality. The analysis, despite coming from a multiethnic major urban hospital system, is still single-center. Vitamin D levels were limited to cross-sectional analysis at one point post-transplant, and routine post-transplant 25(OH)D testing had not begun until 2010–2011, with many patients not receiving testing until years post-transplant, with a mean time-to-bloodwork of 2.81 years. Patients at highest risk for PTDM, especially those transplanted between 1998 and 2010, may have developed DM before vitamin D measurement, and there is potential for informative censoring given the survival bias for patients having 25(OH)D levels drawn years post-transplant. The incident rate over post-bloodwork follow-up was 1.743 events per 100 patient-years, which may be lower than estimates in other cohorts, and represents this informative censoring. We could not assess the concurrent presence or effect of vitamin D supplementation at the time of blood draw, specifically only baseline 25(OH)D levels. Pretransplant indicators of diabetes predisposition such as oral glucose tolerance or impaired fasting glucose were not available for our analysis. Finally, intermethod variability is reported for 25(OH)D assays and may reduce the generalizability of the results.^26,27^

In this study, we have demonstrated the differential effect of various MetS components with PTDM, although further mechanistic learnings are limited by the retrospective nature of this study. Our results indicate a compounding association between 25(OH)D deficiency and MetS burden for PTDM risk. Identifying individuals with both specific, high-yield components of MetS and 25(OH)D deficiency as a high-risk subgroup may enable more targeted preventive strategies. Further dedicated, prospective research is needed to elucidate the underlying mechanisms.

## Data Availability

Partial restrictions to the data and/or materials apply. Anonymized data may be made available upon reasonable request from qualified researchers for non-commercial purposes, and with the approval of local ethics committees. Code for data analysis and visualization is made available for review at https://github.com/malyalar/vitdmets.
